# Match Running Performance Profile and Heart Rate Response in Amputee Soccer Players

**DOI:** 10.3390/ijerph20146357

**Published:** 2023-07-13

**Authors:** Foteini-Vasiliki Panagiotopoulou, Yiannis Michailidis, Athanasios Mandroukas, Aris Mavropoulos, Vasilios Tsimaras, Pantelis T. Nikolaidis, Kosmas Christoulas, Thomas Metaxas

**Affiliations:** 1 Laboratory of Evaluation of Human Biological Performance, Department of Physical Education and Sport Sciences, Aristotle University of Thessaloniki, 57001 Thessaloniki, Greece; fotpanagiotopoulou@yahoo.gr (F.-V.P.); ioannimd@phed.auth.gr (Y.M.); thanmandrou@hotmail.com (A.M.); kchristo@phed.auth.gr (K.C.); tommet@phed.auth.gr (T.M.); 2 Department of Sport Science, National and Kapodistrian University of Athens, 17237 Athens, Greece; mavropoulosaris@gmail.com; 3 Laboratory of Motor Behaviour and Adapted Physical Activity, Department of Physical Education and Sports Sciences, Aristotle University of Thessaloniki, 57001 Thessaloniki, Greece; tsimaras@phed.auth.gr; 4 School of Health and Caring Sciences, University of West Attica, 12243 Athens, Greece

**Keywords:** soccer, heart rate, amputation level, running distances

## Abstract

Introduction: The purpose of this study was (a) to create a profile of the running performances of male amputee soccer players in different speed zones, (b) to investigate the relationship between heart rate (HR) and running distance in two soccer matches, and (c) to study the effect of the level of amputation on the running distance covered during a match. Material and Methods: The participants were male amputee soccer players (*n* = 10, Greek, *n* = 5; Belgian, *n* = 5) who played two international, friendly matches. Distances were measured using a global positioning system, and HRs were recorded using a Polar Team Pro. Results: No differences in the distances covered were observed between participants with different levels of amputation (*p* > 0.05). Nevertheless, there was a trend that participants with a below-the-knee amputation tended to cover longer distances in total (difference +262.3 m, Cohen’s d = 0.40) and in zones 2 (+324.4 m, d = 0.79), 3 (+ 7.1 m, d = 0.65), 4 (+22.7 m, d = 0.43), and 5 (+0.4 m, d = 0.20) and less distance in zone 1 (−207.2 m, d = 0.88). They also tended to perform more accelerations (+3.9, d = 0.89) and decelerations (+4.2, d = 0.87) and had a higher mean HR (+8.4%, d = 2.04) than those with an above-the-knee amputation. Moreover, the mean HR corresponded to 83.3% of the HRmax and did not correlate with distance in any speed zone. Conclusions: It was concluded that the level of amputation might influence running performance and acute physiological response during a soccer match.

## 1. Introduction

Soccer is a particularly popular sport since more than 250 million athletes were registered at the beginning of the 21st century and more than 1.3 billion people worldwide have taken part in it [1]. Soccer appears in several alternative forms as adjustments have been made to the dimensions of the pitch, the ball and its regulations to make it possible for people with disabilities (e.g., blind individuals, people with cerebral palsy and amputees) to take part in the sport [2]. Soccer for amputees appeared in the mid-1980s in North America, particularly in Seattle, USA [3], while in Europe, it emerged in the late 1980s (with the foundation of the World Amputee Football Federation, WAFF) [4]. In amputee soccer, a match takes place with two teams of seven players, with one of them as the goalkeeper. The match has a total duration of 50 min divided into two equal periods of 25 min. The duration of the rest period between halves should not exceed 10 min. Outfield players compete using two crutches and one leg without wearing a prosthesis during the match.

From the first appearance of the sport until the present, several steps have been taken to provide more people with the opportunity to participate in the sport, with the organization of international matches and tournaments. At present, there are amputee soccer teams in 47 countries [3].

Amputee soccer is gaining popularity worldwide among individuals with disabilities. The number of participants in adaptive sports, including amputee soccer, have increased in recent years, which has also resulted in an increase in research on adaptive sports [5]. The electronic databases (EBSCO (SPORTDiscus with Full Text, Academic Search Ultimate, Teacher Reference Center, Health Source: Nursing/Academic Edition, MasterFILE Premier), Web of Science, and PubMed (Medline)) were searched, and we observed that the first relevant study appeared in 2000, while in the last five years, the number of studies has increased [6]. However, the total number of studies investigating amputee soccer remains limited. The impact of participation in amputee soccer on the social and psychological characteristics of the participants has been studied thus far [7,8]. There are also studies dealing with amputee soccer players’ physical abilities and anthropometric characteristics [9,10,11]. Amputee soccer is still developing and has become a point of interest for many researchers since it is a non-Paralympic sport discipline that is applying to enter the Paralympic Games.

The load on amputee soccer players is great as the use of crutches load the back, shoulders and arms [12]. This sport places high demands on stability, balance and core strength [13]. The loads on the athletes’ bodies due to crutch use can cause pain and bring about changes in the players’ behavior on the field [14], as well as negatively affecting their performance during a match [15]. In addition, depending on the position and role of each player in the match, fatigue is different. Previous research has shown that forward players show greater signs of fatigue than midfielders and defenders [11], but also that depending on the height of amputation (above or below the knee), there are different energy costs and different rates of fatigue [16].

Several years ago, an attempt was made to measure the physiological requirements of the sport of soccer for able-bodied soccer players. Understanding the physical requirements of a soccer match is important for improving the training process since appropriate training programs can be designed according to the requirements [17]. In order for players to meet the demands of the match successfully, their physical preparation is a very important factor in constantly meeting the demands of the match [18]. This process was particularly aided by the development of global positioning system (GPS) technologies, with which coaches can monitor the external loads (distances covered, accelerations and decelerations) and internal load (heart rate) in real time. In this way, they can form a running profile of each match position (team formation) and each player individually. This provides the opportunity for personalized and therefore more effective training. Studies in this field are particularly limited with respect to amputee soccer. In one of the first studies in this field, Wilson, Riley and Reilly [19] studied the physiological demands on amputee soccer players from the England national team during the world championship held in Russia. The study evaluated 10 players out of a total of seven matches. The results showed that there were no significant differences in mean heart rate between the first (178 beats/min) and second half (176 beats/min). However, from the heart rate values, researchers concluded that matches are played at a fairly high intensity and require players to respond highly physiologically [19]. In another research study, Simim et al. [11] studied the physical demands of amputee soccer, and they noticed slight differences between the distances covered by the players in the two halves. Also, no differences were observed between halves in heart rate frequency and lactic acid values. In contrast, Maehana et al. [20] studied the running profile and the heart rate function of Japanese amputees during soccer matches. The results of their study showed that the total distance covered in the second half of the match was significantly shorter than that of the first half. However, there were no differences between the distances covered in the high-speed zones. They also observed that the average heart rate of the players during the match corresponded to 96.3% of the HRmax. It is assumed that amputee soccer is classified as a high-intermittent sport with periods of low-, medium- and high-intensity activity. Maximum running speed, accelerations and decelerations along a straight line are fundamental skills in amputee soccer. Regarding amputee running, studies have reported on the physical demands of amputee soccer, including anaerobic performance, sprint performance (an important component of sprinting is the capacity to produce a large propulsive force over a short contact time), strength, balance and locomotor development [10]. This information is required to optimize running performance among amputee soccer players.

Another factor researched in sports movement is the cost of energy. Its systematic observation in match conditions provides information on the internal load placed on soccer players and their nutritional needs [21]. The energy cost of a footballer varies as it depends on the number of different types of movement they make during the match. It can be measured/estimated using instruments that measure oxygen intake and heart rate and GPS systems.

Although the aforementioned studies enhanced our understanding of match running profiles in amputee soccer, it is not clear whether running distances vary by match and level of amputation. From our review of the literature, it is understood that studies concerning the running profiles of soccer players with an amputation are limited. Therefore, any study in this field is useful for the creation of running profiles for these players. Such information about performance correlates would aid professionals working with amputee soccer players to develop optimal training programs tailored to individual players’ needs.

From the above, it seems that there are very few studies examining the running profiles of amputee soccer players. The purpose of this study was to create a match running profile for soccer players with an amputation in different speed zones, as well as to investigate a possible correlation of their HRs with the above running distances during two soccer matches and to investigate the effect of the level of amputation on the match running distance covered during the race. As mentioned above, the creation of the profile will aid personalized exercise and therefore help to achieve the players’ best performances. Also, the study of heart rate in relation to the running speed zone will help control the intensity of training for soccer players. Finally, it will be observed whether the level of amputation affects the running profile, which could affect the choice of players on a team if it is a limiting factor.

## 2. Materials and Methods

### 2.1. Participants

The current study involved ten amputee soccer players (Greek, n = 5; Belgian, n = 5) aged 25–48 years. The goalkeepers were required to be single-arm amputees and were excluded as their disability was limited only to the upper limbs and they did not play in both matches. Of the 10 participants, 5 had an amputation below the knee and 5 above it. The years of their coaching experience ranged from 2 to 7 years, while the training frequency of the players was 2 times per week with a duration of 2 h. All participants were informed of the benefits and potential negative effects of their participation in the study and signed consent forms for their participation.

The participants were told not to consume any meals 3 h prior to the matches. The experiment complied with the ethics criteria of the Research Code of the Aristotle University of Thessaloniki, in accordance with the ethical standards of the Helsinki Declaration. The characteristics of the participants are presented in Table 1.

### 2.2. Procedures

The measurements were obtained during two international, friendly matches between the Greek and Belgian national teams. The matches took place during a three-day period on natural turf. The matches were held according to the rules of the sport. Accordingly, two halves were played (2 × 25 min) on a smaller pitch (from 60 × 35 m) by seven players (six field players and one goalkeeper). Single-leg amputees (either above or below the knee) played without prostheses, on aluminum wrist crutches (field players).

The two international, friendly soccer matches took place in the afternoon at the end of the season and were two days apart. The temperature was 21 ± 2 °C, while the relative humidity was at 60 ± 4%.

The players of both teams arrived at the stadium 60 min before the start of the match. After the instructions were provided by the coaches, the players put on their GPS devices and went out onto the field 30 min before the start of the match to complete a 20-min warm-up. They followed this with hydration, and the match began. Each player wore the same GPS transmitter during both matches. The GPS transmitter was placed in a special position on the undershirt on the upper back of the player.

Global positioning system (GPS) receivers (10 Hz; STATSports, Newry, UK) were used to measure the running distances. The receivers were placed before the two teams warmed up in special vests. This system has been validated against a kinematic video analysis of shuttle runs of various speeds and distances [22] and ground distances of 20 m, 128.5 m and 400 m [23]. In addition, each player wore an HR monitor to record their HR (Polar Team Pro, Kempele, Finland). The HR monitor’s reliability in measuring distances has been established in the past [23]. The speed zones designated for the study were based on the research of Maehana et al. [20] and were zone 1, standing (<0.4 km/h); zone 2, walking (0.4–5 km/h); zone 3, low-speed running (5–8 km/h); zone 4, moderate-speed running (8–13 km/h); zone 5, high-running speed zone (13–18 km/h); and zone 6, sprinting (≥18 km/h). In addition, high-intensity running (HIR) was defined as a speed of over 13 km/h. After the end of each match, the data from the GPS transmitter and the HR monitor were transferred to a computer using appropriate software.

### 2.3. Statistical Analysis

For statistical analysis, Statistical Software SPSS v.24.0 was used. The results were presented as means ± SDs. A descriptive statistic was provided out for all variables. The Kolmogorov–Smirnov test established the normal distribution of the sample. An independent samples *t*-test was used to compare the results regarding the level of amputation. In addition, possible correlations were tested using a Pearson correlation analysis. The magnitudes of differences (Cohen’s d) were evaluated using the Hopkins scale as follows: 0–0.2, trivial; 0.2–0.6, small; 0.6–1.2, moderate; 1.2–2.0, large; and >2.0, very large [24,25]. The level of significance was set to *p* < 0.05.

## 3. Results

The anthropometric characteristics of the ten players are shown in Table 1.

Of the ten participants, five had an amputation above the knee and the rest had an amputation below knee level. The results of the statistical analysis showed that there were no differences between the players’ anthropometric characteristics in relation to the level of amputation. Table 2 shows the characteristics of the participants in relation to the level of amputation.

The soccer players with an amputation above the knee were 4.6 years younger, 6.5 kg lighter and had 8.4% lower BMI values. However, differences were observed in the %HRmax in which they competed (average HR/Hrmax), where players with a below-the-knee amputation had higher values (t = 3.322, *p* = 0.011). The values of their HR indicators are presented in Table 3.

**Figure 1:**
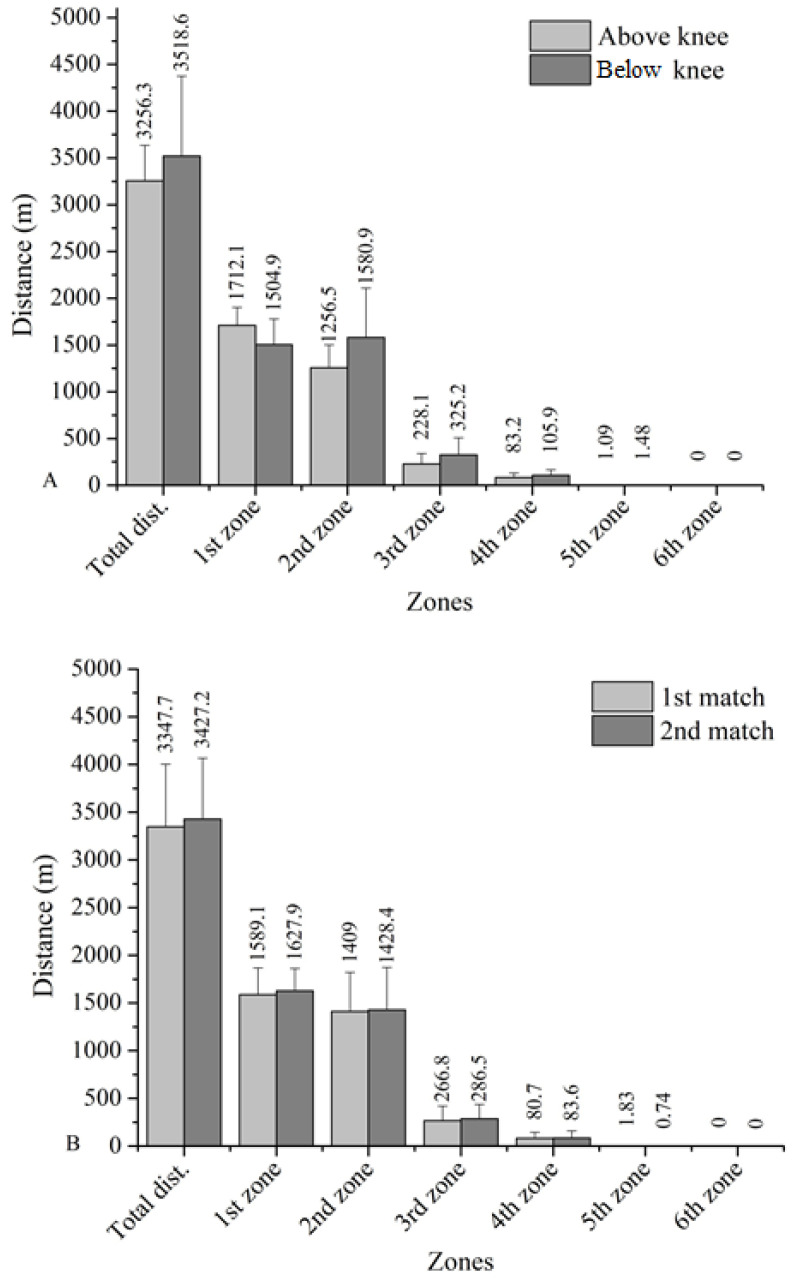
Match running performance differences between (**A**) level of amputation and (**B**) two matches.

The distances covered by the players in the two matches did not differ in terms of the level of amputation (t = 0.628, *p* = 0.548). Also, the distances covered in the different speed zones did also not differ between the two matches. The distances covered by the players in terms of the level of amputation between the two matches are presented in Figure 1 (Figure 1A, Figure 1B respectively).

**Figure 2:**
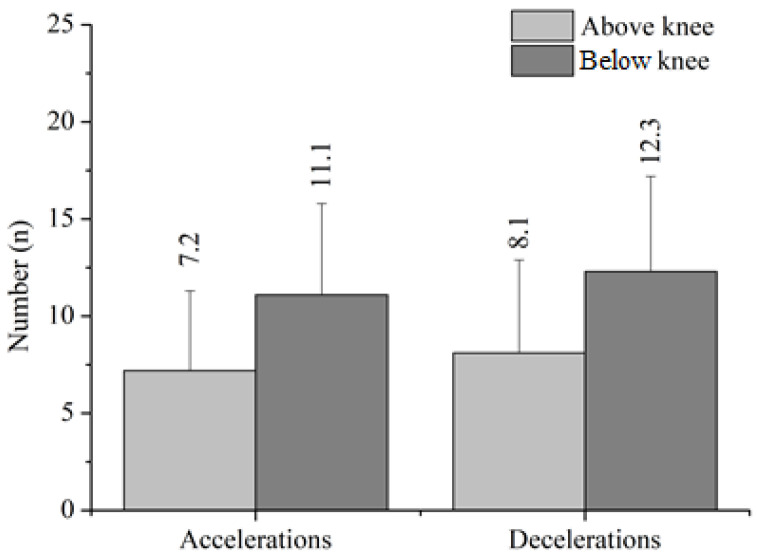
Acceleration and deceleration differences between the levels of amputation.

Players with a below-the-knee amputation covered an 8.1% further distance than players with an amputation above the knee (but no statistically significant differences appeared). Players with an above-the-knee amputation tended to cover more meters in zone 1 (zone 1 by 14%), while players with a below-the-knee amputation covered more meters in zones 2, 3, and 4 (zone 2 by 25.8%, zone 3 by 42.6% and zone 4 by 27.3%) and performed more decelerations (51.9%). In Figure 2, the accelerations and decelerations in relation to the level of amputation are presented. There was a trend that participants with a below-the-knee amputation tended to cover longer distances in total (difference +262.3 m) and in zones 2 (+324.4 m), 3 (+97.1 m), 4 (+22.7 m) and 5 (+0.4 m) and less distance in zone 1 (−207.2 m). They also performed more accelerations (+3.9) and decelerations (+4.2) and had a higher mean HR (+8.4%) than those with an above-the-knee amputation.

**Figure 3:**
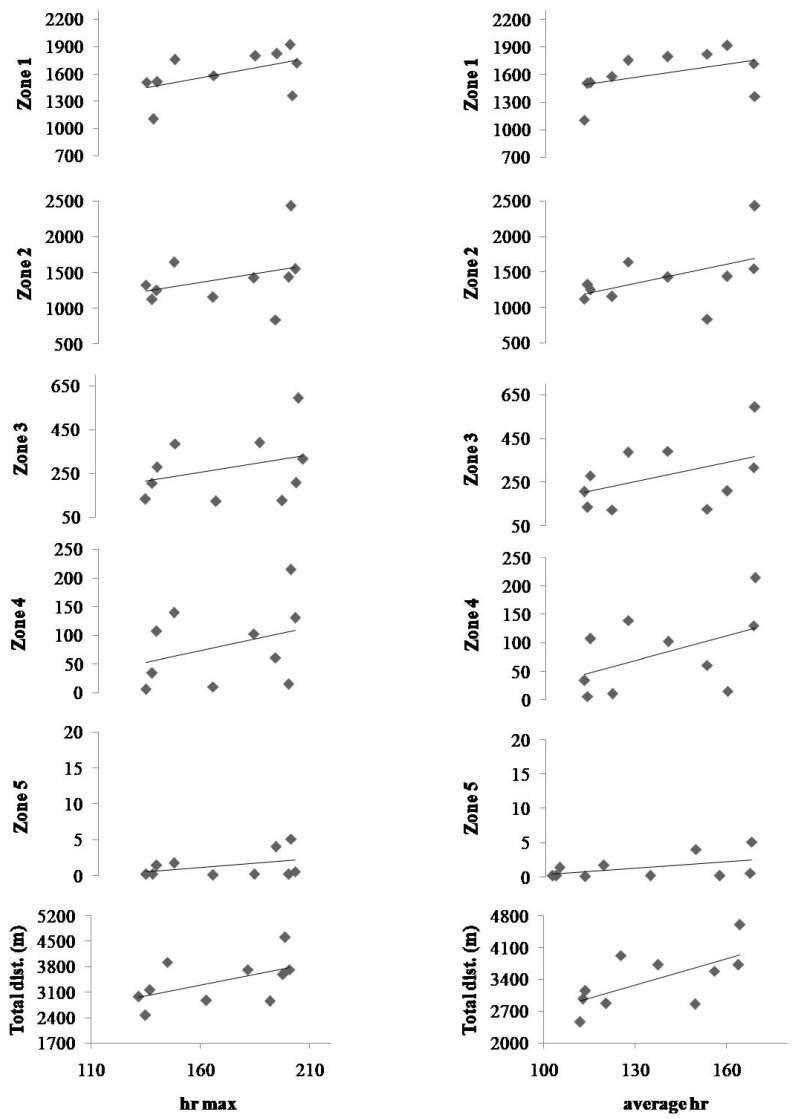
Correlations of maximal (**left**) and average heart rates (**right**) with distances covered in particular zones. Grey symbols denote individual values, whereas lines represent the linear relationship between the variables.

The soccer players covered slightly longer distances in the second match, both in total and in most zones separately, but without a statistically significant difference. The correlation analysis showed no correlations between HR indexes and the distances covered during matches. The correlations are presented in Figure 3, and the statistical indicators of the correlations of maximum and average heart rates with distances in the different speed zones are presented in Table 4.

## 4. Discussion

### 4.1. Heart Rate

The purpose of this study was to create running profiles of soccer players with amputations in different speed zones, as well as to investigate a possible correlation of their heart rates (HRs) with the above running distances during two soccer matches. Another aim of the study was to investigate the effect of the amputation level on the match running distances covered during the match.

The results showed that the average maximum heart rate of the players participating in the two matches was 168 ± 28 b·min^−1^, while the average heart rate was 140 ± 20 b·min^−1^. The average HR was 83.3% of the HRmax, and our results are similar with a previous study [26] that mentioned values of %HRmax near 88%. The soccer players in our study competed at a lower intensity in comparison with previous studies [11,20,27], which mentioned values near 95% of the HRmax. The results of this study clearly show that the findings can serve as guidance, helping soccer players with amputations and their trainers reach maximal performance. Also, the results showed no correlations between maximum and average heart rates with the distances covered in different speed zones. Although we hypothesized that there would be a positive correlation of the distance in high-speed zones with the mean heart rate, this was not observed. This may be due to the fact that the soccer players in this study covered only a few meters in the high-speed zones.

### 4.2. Level of Amputation and Heart Rate

Regarding the level of amputation, it was found that soccer players with a below-the-knee amputation tended to exercise at a higher intensity, as indicated by their higher mean HR (as a percentage of their HRmax) than those with an amputation above the knee. This finding is in agreement with the running performances, in which the soccer players with a below-the-knee amputation covered more distances, except in zone 1, than those with an amputation above the knee. In their study, Vllasoli et al. [28] reported that energy costs during physical activities were higher for players with an above-the-knee amputation than in players with an amputation below the knee. Moreover, Van Schaik et al. [29] mentioned that while walking, oxygen uptake and HR increased as walking speed and amputation level increased. In our study, soccer players with a below-the-knee amputation competed with higher HRs than those with an above-the-knee amputation. This observation may be due to the fact that they tended to run further distances than the others. There are no studies to investigate changes in HR in relation to the level of amputation in soccer. In summary, the distances covered, in total as well as in most of the speed zones, the number of accelerations and decelerations and the mean HR reflected more active profiles of the soccer players with an amputation below the knee.

### 4.3. Running Distances

The results of the study showed that the total distance covered by the players in both matches was 3387.5 ± 638.1 m. These values were greater than those reported in previous studies [20,27] and range in 2984.2 ± 561.6 m. However, in another study, Simim et al. [11] reported that the total distance covered by their participants was close to 6 km. This finding contrasts with all previous studies, which indicated values close to 3500 m. Despite the importance of sprint performance in amputee soccer [30,31], the distance covered during HIR was 1.28 ± 1.86 m. This measurement indicates that almost no player ran at a speed of more than 13 km/h. In contrast, Maehana et al. [20] reported that Japanese soccer players covered about 205 m, while Simim et al. [11] reported that Brazilian soccer players covered about 126 m during HIR. It can be inferred that in this study, the soccer players competed with less intensity by covering more distance in low-speed zones and less distance in high-speed zones compared to the above-mentioned studies. The above researchers concluded that in order to increase the distance covered during high-intensity running (HIR ≥ 13 km/h), players should increase and improve their interval training. Perhaps the type of training undertaken by the players in this study, the differences in the characteristics of the participants (training experience) and the level of difficulty of the league matches affected the above parameters. In amputee soccer, as in able-bodied soccer, high-intensity actions (e.g., sprinting) determine the outcome of a match. Therefore, the application of training methods that will help increase the distances covered at high-intensity speeds will most likely help improve the performances of amputee soccer players.

Considering the two matches, all athletes covered similar distances. Also, players with a below-the-knee amputation tended to cover a longer distance on average than players with an above-the-knee amputation (8.1%). There is no research relating to soccer regarding the level of amputation and match running performance. More research with larger samples is needed to clarify this question. The tactics and the position role have an impact on the match running performance [32,33].

The study also has some limitations. Initially, the study sample was limited, so we could not generalize the findings of the study. Also, the matches in this study belong to the category of “international friendly matches”, which may have affected the results. Finally, the number of matches was too small to draw firm conclusions. However, studies in this field are minimal, and in the future, studies with larger samples, in competitive championships and with more matches are required.

## 5. Conclusions

This study offers better insight in terms of classification for the sport amputee soccer. There is an indication that providing one sport class among the outfield players was accurate regarding the repeatability of the findings between the two soccer matches. However, there should be more studies with larger samples that examine more aspects of the discipline to achieve a more reliable classification system. Furthermore, the players with an amputation below the knee exercised at higher intensities. The lack of research data in soccer on performance in relation to level of amputation did not allow us to generalize our conclusions. Finally, given the lack of coverage of distances at speeds above 13 km/h, and taking into account conclusions of previous studies, we can consider that the importance of the matches and the training backgrounds of the athletes may have influenced this factor. It is well known that in the soccer training process, special emphasis is placed on the intensity of training. Coaches want a high level of intensity in most weekly microcycle workouts as players will play similarly to the way they trained, and coaches should be obliged to evaluate the motor performance of their players to notice progress or weaknesses in the training process. As mentioned above, heart rate control during workouts is the most common means of controlling intensity during training, especially at present, when heart rate technology is accessible to everyone. It is likely that increasing intensity during training for the above amputee soccer players will help them compete at higher intensities.

## Figures and Tables

**Table 1:** Participants’ characteristics (mean ± standard deviation).

	Total
Number (n)	10
Age (years)	33.5 ± 6.3
Training experience (years)	3.6 ± 2.4
Height (cm)	177.7 ± 6.6
Weight (kg)	78.45 ± 13.1
BMI (kg·m^−2^)	24.7 ± 2.8
Amputation (above/below knee)	5 and 5

BMI: body mass index.

**Table 2:** Participants’ characteristics by level of amputation (means ± SDs).

	Amputation	
	Below Knee	Above Knee
Number (n)	5	5
Age (years)	35.8 ± 4.8	31.2 ± 7.3
Training experience (years)	4.0 ± 2.7	3.2 ± 2.3
Height (cm)	178.0 ± 4.3	177.4 ± 8.9
Weight (kg)	81.7 ± 9.3	75.2 ± 16.5
BMI (kg·m^−2^)	25.7 ± 2.1	23.7 ± 3.2

BMI: body mass index.

**Table 3:** Heart rate changes during matches in relation to the level of amputation (means ± SDs).

	Above Knee	Below Knee
AVG HR (b·min^−1^)	146.7 ± 7.1	159.6 ± 10.5
HRmax (b·min^−1^)	188.9 ± 7.2	191.3 ± 17.6
%HR_max_	77.7%	83.4%

**Table 4:** Results of the Pearson correlation test for heart rate and distances covered in different speed zones.

	HRmax	HRaverage
Total Distance	r = 0.537	r = 0.651
	*p* = 0.110	*p* = 0.052
Zone 1	r = 0.513	r = 0.446
	*p* = 0.130	*p* = 0.197
Zone 2	r = 0.336	r = 0.482
	*p* = 0.343	*p* = 0.158
Zone 3	r = 0.327	r = 0.446
	*p* = 0.356	*p* = 0.196
Zone 4	r = 0.339	r = 0.475
	*p* = 0.337	*p* = 0.165
Zone 5	r = 0.380	r = 0.473
	*p* = 0.279	*p* = 0.168

## Data Availability

All data are available by the corresponding author upon reasonable request.
